# Cortical gyrification predicts initial treatment response in adults with ADHD

**DOI:** 10.1038/s41398-025-03681-0

**Published:** 2025-10-17

**Authors:** Jonathan Laatsch, Frederike Stein, Simon Maier, Swantje Matthies, Esther Sobanski, Barbara Alm, Ludger Tebartz van Elst, Axel Krug, Alexandra Philipsen

**Affiliations:** 1 Department of Psychiatry und Psychotherapy, University Hospital Bonn, Bonn, Germany; 2 Department of Psychiatry und Psychotherapy, University of Marburg, Marburg, Germany; 3 Department of Psychiatry and Psychotherapy, Medical Center – University of Freiburg, Faculty of Medicine, University of Freiburg, Freiburg, Germany; 4 Department of Child and Adolescent Psychiatry Lucerne, Lucerne, Switzerland; 5 Department of Psychiatry and Psychotherapy, Central Institute of Mental Health, Medical Faculty of Mannheim, University of Heidelberg, Mannheim, Germany

**Keywords:** Neuroscience, ADHD

## Abstract

While the need for personalised treatment approaches grows in recognition, predicting treatment outcomes for adults with Attention-Deficit/Hyperactivity Disorder (ADHD) remains underexplored. Recent interest has turned to the brain’s surface and its association with treatment response. Although the precise interplay between cortical gyrification and ADHD treatment outcomes remains to be elucidated, preliminary investigations suggest a promising avenue for diagnostic innovation. Expanding upon the Comparison of Methylphenidate and Psychotherapy in Adult ADHD Study (COMPAS), we investigated the prognostic value of cortical gyrification in predicting treatment response. Specifically, we explored how pre-treatment cortical gyrification might predict response to psychotherapy or clinical management in combination with either methylphenidate or placebo following a 12-week intensive treatment period. Cortical gyrification was assessed using 121 T1-weighted anatomical scans. Linear regression models investigated the predictive value of cortical gyrification, regressing baseline cortical structure against post-treatment severity. All brain structural analyses were conducted using the threshold-free cluster enhancement (TFCE) approach and the Computational Anatomy Toolbox (CAT12) within the Statistical Parametric Mapping Software (Matlab Version R2021a). Results revealed significant positive region-specific associations between cortical gyrification and treatment response across three symptom dimensions, with significant associations localised predominantly in frontal regions of the left hemisphere. Our findings emphasise that increased cortical gyrification in frontal cortical regions signifies enhanced treatment efficacy following a 12-week intervention. Further research in this area is imperative to verify the reliability of biological markers in view of treatment success to potentially reduce unnecessary drug-related side-effects, minimising delay from receiving more effective treatments, and increase treatment adherence.

## Introduction

While historically considered a neurodevelopmental disorder confined to childhood, Attention-Deficit/Hyperactivity Disorder (ADHD) persistently impacts up to 60% of individuals into adulthood [1, 2]. Characterised by its intrinsic heterogeneity, ADHD manifests itself across a spectrum of severity among those affected. Patients present distinct manifestations of symptom domains, aetiologies, developmental trajectories, and response to treatment [3, 4].

Effective management of ADHD in adults often necessitates a multifaceted approach, encompassing both pharmaco- and psychotherapeutic interventions. Although the administration of stimulants (e.g., methylphenidate) and nonstimulants (e.g., atomoxetine) has been demonstrated to reduce core ADHD symptoms in the short term [5–8], the effectiveness of pharmacotherapy varies considerably. Approximately 30% of patients do not respond to pharmacotherapy and the inter-individual variability in tolerability of associated side effects often results in discontinuation of medication [9, 10]. Where patients respond, the benefit of pharmacologic treatment may decline over time [11, 12], demonstrating the necessity of other strategies to improve the functional outcomes of patients.

Alongside pharmacotherapy, numerous nonpharmacological interventions have been introduced for adults with ADHD. Among others, these approaches include cognitive behavioural therapy (CBT), psychoeducation, emotional regulation therapy, mindfulness therapy, and neurofeedback [13]. Nevertheless, previous systematic reviews and meta-analyses on the efficacy of psychotherapy on ADHD core symptoms [14–16], internalising symptoms [17, 18] and emotional regulation [19] yielded inconclusive results with either small or modest effects due to limited sample size or methodology (i.e., open-label studies). Recently, however, a meta-analysis by Liu et al. [20] focusing on previous methodological shortcomings arrived at a more definitive conclusion regarding the effectiveness of CBT in adults with ADHD. First, CBT significantly reduced the severity of both core ADHD symptoms and emotional symptoms, with medium to large effect sizes. Second, ADHD severity total scores, inattention and hyperactivity/impulsivity symptoms were significantly lower after CBT. Third, the reduction in ADHD symptoms correlated with an improvement of quality of life. Together, these findings suggest that CBT is a promising strategy for adults with ADHD in its ability to offer symptom remission and improved life quality.

In the light of precision medicine, there have been only few studies aiming to predict treatment success in ADHD [21]. For this reason, there is currently no clear evidence to guide decision making about who will tolerate and/or respond best to which treatment approach. Presently, it is not possible to predict course or outcome at the level of the individual patient [22]. The inherent complexity and diversity within ADHD pose significant challenges to predicting treatment efficacy. Still, some research groups tried to detect biological predictors of psychostimulant treatment response in adults affected by ADHD [23–26]. Among the findings that significantly predicted treatment response to psychostimulants were genetic markers [27], task-based- [28] and structural neuroimaging markers [25], neurophysiological- [29] as well as electrophysiological markers [30], constructing a complex and diverse clinical picture [23]. Even though these results may be promising for improving diagnostic accuracy, additional research will have to verify the reliability of the different biological markers in view of the complex pathophysiology of ADHD [23].

While attempts have been made to predict treatment response to pharmacotherapy, research investigating biological predictors of treatment response to psychotherapy in adults with ADHD has yet to begin. This is despite substantial evidence to suggest that such predictors may indeed exist. Psychological interventions have the capacity to modify brain function across various psychopathological conditions [31]. First, research suggests that changes in brain function following psychotherapy correlate with symptom improvement [32]. Second, psychotherapy can lead to normalisation of brain structure and function in patients with conditions such as obsessive compulsive disorder, depression, or schizophrenia [33–35]. Third, comparisons between neurobiological changes post-psychological treatment and those following pharmacological treatment reveal that psychotherapy can have effects on brain function comparable to medication, though not universally across all disorders [31]. While direct evidence may be lacking, the existing body of research strongly implies a significant relationship between psychotherapy and neurobiological changes, highlighting the potential for further investigation in this area.

Recently, there has been increasing interest toward exploring the brain surface as predictor of treatment outcomes across a variety of psychiatric conditions [36–38]. Especially local cortical gyrification and the overall gyrification index (GI) have received most attention. Nevertheless, research investigating the GI in relation to ADHD in adulthood are notably scarce and yielded inconclusive results. Ambrosino et al. [39] found decreased cortical folding in the left rostral middle frontal cortex and right pars opercularis, and Mous et al. [40] reported increased gyrification in the left medial temporal lobe. Contrastingly, neither Forde et al. [41], Shaw et al. [42] and Gharehgazlou et al. [43], nor our research group [44] report differences in either global or local gyrification between patients with ADHD and healthy controls. Such inconsistencies likely reflect both methodological variability and sample heterogeneity. Differences in imaging protocols, including the choice of smoothing parameters, and whether gyrification is assessed local or globally can substantially influence outcomes. Moreover, variability in sample characteristics such as age distribution, comorbidity profiles, ADHD subtypes, and prior treatment exposure may further contribute to variability across studies. Although the general consensus holds that the GI remains relatively stable after the third trimester of pregnancy [45], more recent work by White et al. [46] propose that the GI may also undergo significant modulation during adolescence and maturity into early adulthood. In this light, cortical gyrification may not only reflect early neurodevelopmental processes, but also serve as a dynamic marker with potential utility in predicting treatment response. In fact, there have been prognostic studies on the relationship between cortical gyrification and psychotic disorders predicting treatment response [36, 37], subsequent recurrence in patients with first-episode schizophrenia [47] or conversion to psychosis [48, 49]. Studies in adults with ADHD, however, are yet to be conducted but may hold promising diagnostic value.

Based on the considerations given above, the present study investigated whether alterations in cortical gyrification hold prognostic value in predicting treatment response in adults with ADHD. Expanding upon our prior work [50] within the framework of the Comparison of Methylphenidate and Psychotherapy in Adult ADHD Study (COMPAS) cohort, we explored cortical gyrification in relation to treatment response following a 12-week intervention period, both across the entire sample and within each treatment group. COMPAS was the first, and so far largest, multi-centre randomised clinical study that evaluated the effects of group psychotherapy (GPT) compared with clinical management (CM) combined with methylphenidate (MPH) or placebo (PLA) in adults with ADHD [51–53]. In pursuit of adequately addressing the complexity of neuroanatomical variability, we applied the threshold-free cluster enhancement (TFCE) approach. By addressing key limitations of voxel-based morphometry, TFCE enables the detection of both spatially diffuse and sharply localized structural changes while maintaining strict control over family-wise error [54]. Its sensitivity to nuanced structural patterns has solidified its utility in capturing neuroanatomical variability, particularly in studies of neurodevelopmental disorders [55]. Within this methodological and clinical framework, our objective offers the potential to discern individuals prone to suboptimal treatment responses before the commencement of MPH prescription or the initiation of psychotherapeutic interventions. Such initiatives bear clinical significance to reduce unnecessary drug-related side-effects, minimising delay from receiving more effective treatments, and patient discouragement. Given the limited research available on predicting treatment response in adults with ADHD, encompassing both pharmacological- and psychotherapeutic intervention in relation to cortical gyrification, we will explore gyrification without predefined hypotheses. Nonetheless, based on existing research in schizophrenia [36, 37], we anticipate a positive relationship between treatment response and cortical gyrification.

## Methods and materials

### Ethical considerations

The study obtained ethical approval from the Ethics Commission of the University Medical Center Freiburg (Approval ID: 217/06) and additional authorisation from the German authorities for pharmacological trials (EudraCT No.: 2006-000222-31), adhering to the principles outlined in the Declaration of Helsinki [56]. Subsequently, the trial was registered with Current Controlled Trials (ISRCTN54096201). Prior to participation, all individuals provided written informed consent. Details of the eligibility criteria for study participation can be found in the supplemental material, Table S1. Patient recruitment took place between January 2007 and August 2010, with the study treatment concluding in August 2011.

### Treatment groups

The COMPAS trial employed a four-arm, randomised, multicentre design to investigate the efficacy of psychotherapy and pharmacological treatment in adults with ADHD. Diagnoses were confirmed via expert psychiatric assessment and validated using structured clinical interviews and rating scales. A total of 419 participants were randomized into one of four treatment conditions: (1) GPT & MPH, (2) GPT & PLA, (3) CM & MPH, or (4) CM & PLA. GPT followed a manualised, evidence-based protocol, delivered weekly for 12 weeks and monthly thereafter for 10 additional sessions. CM served as an active control condition simulating routine psychiatric care of nondirective supportive counselling on the basis of individual sessions lasting 15–20 min. Methylphenidate was titrated over six weeks up to a maximum of 60 mg/day. Treatments were assigned in a 1:1:1:1 ratio, with blinding for medication and open allocation for GPT vs. CM. Observers rating symptom outcomes were blinded to treatment allocation. Treatment fidelity was ensured through session recordings and independent expert reviews.

### Participants

Building on the foundation of our prior publication [50], we conducted this study with the identical cohort of research participants previously outlined in detail.

Among the 196 patients who consented to the imaging study, 46 were excluded prior to image acquisition due to withdrawal of consent, noncompliance, discontinuation of study participation, scheduling difficulties, claustrophobia, or the presence of contraindicating implants. Hence, T1-weighted images of 150 patients were acquired. Nevertheless, among the initial 150 patients who granted consent for the imaging study, only 123 continued the study until week 13. Subsequently, one patient was retrospectively excluded for above threshold psychometric documentation on the main assessment of ADHD symptomatology (e.g., outlier). Another patient was excluded to ensure homogeneity of MRI data quality. The final sample available for analysis consisted of 121 MRI data sets. Within this sample, 58 patients were treated with MPH while 63 received PLA, with 59 undergoing GPT and 62 enrolled in CM. Figure S1 displays the flowchart of participant selection (see Supplemental Material). Table 1 summarises the demographic characteristics of the full sample. Tables S2–4 address psychometric data as well as group differences at baseline and treatment effects over time (see Supplemental Material).

**Table 1:** Demographic and psychometric data for the full patient sample.

Age (years)	
Mean ± SD (*n*)	35 ± 9.86 (121)
Range	19–58
Sex	
Male	50.4% (61/121)
Verbal IQ, mean (SD) [range]	
Mean ± SD (*n*)	113 ± 15.6 (121)
Range	88–145
TIV, mm^3^	
Mean ± SD	1453.31 ± 143.22
Education: university entrance diploma, y 5-12/13	53% (64/121)
WURS-k score	
Mean ± SD (*n*)	39.76 ± 8.56 (121)
Range	29–76
ADHD subtype	
Combined	52% (63/121)
Predominantly inattentive	45% (55/121)
Predominantly hyperactive-impulsive	3% (3/121)
Previous psychopharmacological treatment	
≥1 Previous psychopharmacological medication	50% (61/121)
Antidepressants	32% (39/121)
Methylphenidate, amphetamine, or other psychostimulants	25% (30/121)
Sedatives, neuroleptics, atomoxetine hydrochloride, mood stabilizers, or others	12% (14/121)

*TIV* total intracranial volume, *WURS-k* Wender-Utah rating scale.

### Assessment of symptomatology

ADHD-related symptomatology was assessed using the observer-rated German version of the Conners’ Adult ADHD Rating Scale (CAARS) [57]. The questionnaire contains 66-items that are graded on a Likert scale from 0 (not at all) to 3 (severely). It is available in both, a self-report and observer-rated form (CAARS S:L/ CAARS O:L) and addresses four primary factors of symptoms: inattention/cognitive problems, hyperactivity/restlessness, impulsivity/emotional lability, and issues with self-concept [58]. For more information regarding the CAARS questionnaire, cf. [58–60]. For this study, we chose to focus primarily on the CAARS O:L to assess treatment response in order to avoid response- or social desirability biases.

### MRI data acquisition and Pre-processing

A detailed description of the MRI data acquisition and pre-processing can be found in our prior work [50]. Briefly, MRI scans were solely performed at the study centre located in Freiburg. MRI data were preprocessed using the default parameters integrated in the CAT12 Toolbox (Computation Anatomy Toolbox for SPM, build 12.7 r1720, http://dbm.neuro.uni-jena.de/cat/), building on SPM12 v7771 (Statistical Parametric Mapping), providing bias-corrected, tissue classified, and normalised data ratings. Images were spatially registered, segmented, and normalised using a DARTEL algorithm [61–63]. GI parameter estimation involved fully-automated methods projecting local maxima to other grey matter voxels using a neighbour relationship defined by the white matter distance [64]. Quality control of processed data was applied as implemented in CAT12, controlling for noise, inhomogeneity and resolution. Subsequently, the T1-MRI data sets were spatially smoothed using a Gaussian kernel with a full width at half maximum of 20 mm.

### Statistical analyses

Demographic and psychometric data were obtained using SPSS software, version 27 (IBM Corp., Armonk, New York, USA). Separate multivariate analyses using one-way MANCOVAs were used to assess baseline symptomatology. Baseline effect analyses defined the subscales of the CAARS O:L as dependent variables, with the treatment condition (e.g., GPT/ CM, MPH/ PLA) as independent variable. Group- and time effects were investigated using separate general linear model repeated measures analyses of covariance incorporating within-subject factor ‘Time’ and between-subject factor ‘Treatment’. Here, the dependent variables also corresponded to the subscales of the CAARS O:L. Both, ‘Treatment’ and ‘Time’ included two levels: ‘Treatment’ was defined by the specific treatment condition, and ‘Time’ reflected baseline versus post-treatment scores after 12 weeks. Age and sex served as covariates in all analyses. Post hoc analyses were conducted where appropriate to further explore significant findings. All analyses were corrected for multiple comparisons using Bonferroni adjustments and significance was set to *p* < 0.05.

For the primary analyses, all brain structural analyses using T1-weighted images were performed using the CAT12 toolbox for SPM12 Version 7771 under Matlab Version R2021a (MathWorks, Natick, Massachusetts, USA). We used separate linear regression analyses to investigate correlations between treatment response and the GI, both for the whole sample and at a subgroup level. Treatment response was operationalised by computing the change between the scores across the eight subscales of the CAARS O:L of week 0 and week 13. For the subgroup analyses, the total sample was divided into two treatment groups based on the active treatment condition (MPH or GPT). The groups were designed to account for variables not of primary interest, effectively cancelling out their influence and enabling a focused evaluation of the specific treatment effects. Age and sex were controlled for by including them as covariate of no interest. Absolute threshold masking was set to a threshold value of 0.1. Subsequently, TFCE with 5000 permutations for GLM contrast generation was utilised for further analysis, testing for both positive and negative correlations. Clusters identified in the surface parameter analyses were labelled using the Desikan-Killiany atlas [65]. Statistical significance was determined at cluster threshold extend of *k* > 35 and *p* < 0.05 family wise error (FWE)-corrected (combined peak-cluster-level), controlling for multiple comparisons.

## Results

### Total sample

**Fig. 1:**
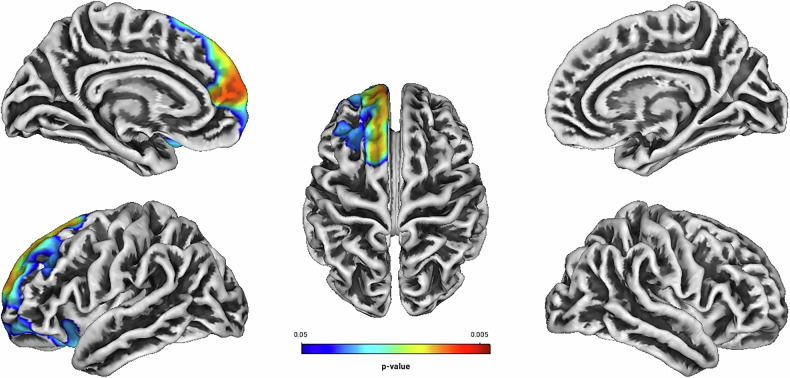
Positive association between cortical gyrification and CAARS O:L Total Score Change (Weeks 0–13). Cluster of significant positive correlation at p < 0.05 after TFCE, FWE-corrected combined peak-cluster level.

**Fig. 2:**
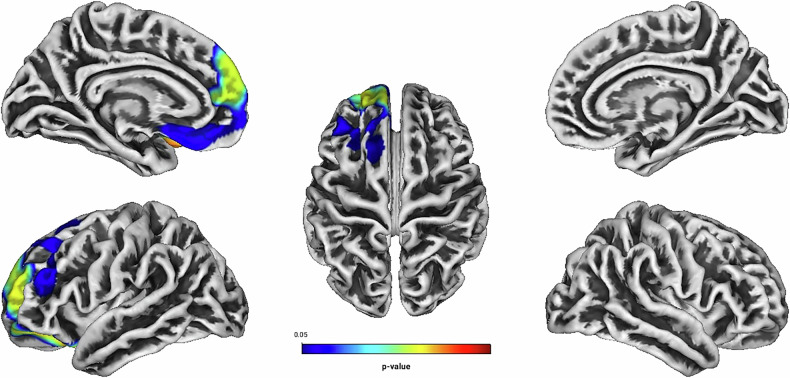
Positive association between cortical gyrification and CAARS O:L Hyperactivity Change (Weeks 0–13). Cluster of significant positive correlation at p < 0.05 after TFCE, FWE-corrected combined peak-cluster level.

**Fig. 3:**
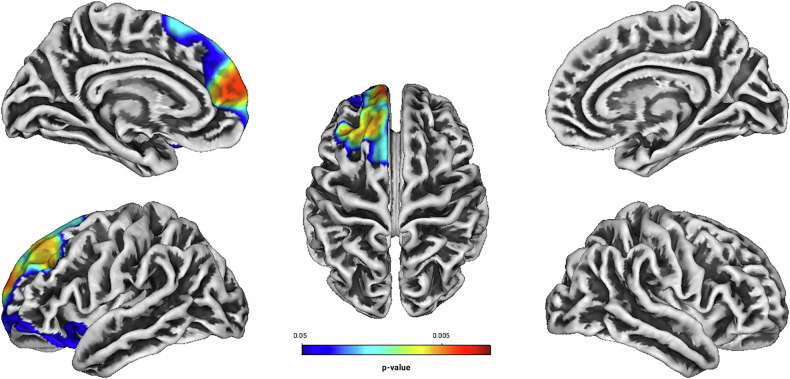
Positive association between cortical gyrification and CAARS O:L Self-Concept Change (Weeks 0–13). Cluster of significant positive correlation at p < 0.05 after TFCE, FWE-corrected combined peak-cluster level.

**Fig. 4:**
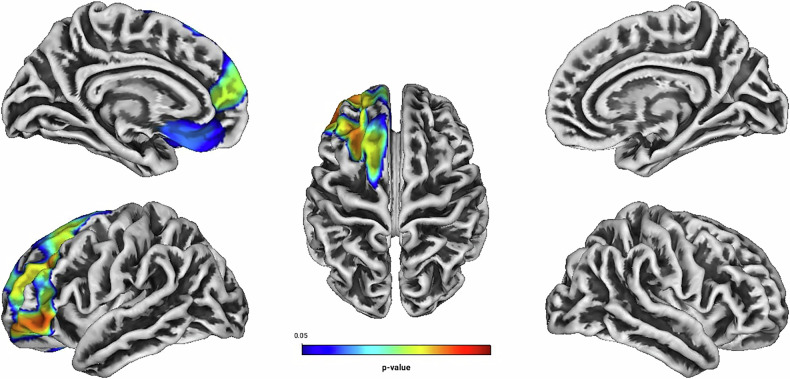
Positive association between cortical gyrification and CAARS O:L DSM Hyperactivity/Impulsivity Change (Weeks 0–13). Cluster of significant positive correlation at p < 0.05 after TFCE, FWE-corrected combined peak-cluster level.

**Fig. 5:**
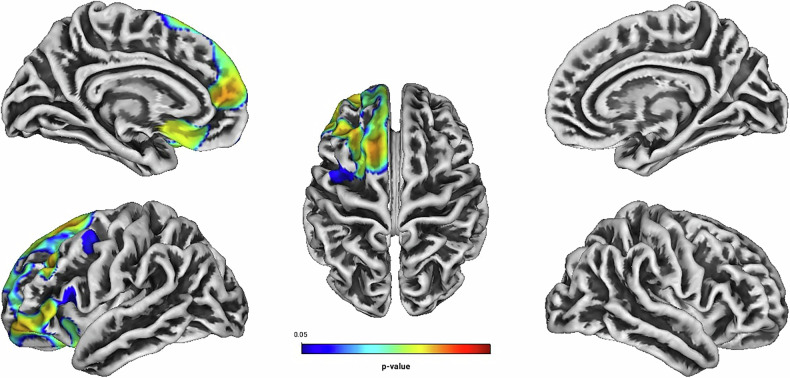
Positive association between cortical gyrification and CAARS O:L DSM Total Score Change (Weeks 0–13). Cluster of significant positive correlation at p < 0.05 after TFCE, FWE-corrected combined peak-cluster level.

Independent of treatment modality, linear regression analyses showed positive associations between Total Score, Hyperactivity, Self-Concept, DSM Hyperactivity/Impulsivity, DSM Total Score and the GI located predominantly in frontal structures of the left hemisphere (see Table 2 and Figs. 1–5). There were no statistically significant correlations between Inattention, its DSM index, or Impulsivity and the GI [all associated *TFCE* values < 5982.85, FWE-corrected peak-cluster level *p* > 0.088].

**Table 2:** Associations of cortical gyrification index and changes in ADHD-related psychopathology (Weeks 0–13), measured by the CAARS O:L for n = 121, at p < 0.05, TFCE, FWE-corrected combined peak-cluster level.

Factor	Corre-lation	Coordinates of the maximum intensity voxel (x/y/z) MNI	Anatomical labelling	Hemisphere	*k*	*p*
Total Score	positive	0/105/33	46% Superior frontal 25% Rostral middle frontal 17% Lateral orbitofrontal	Left	2451	0.007^a^
Hyperactivity	positive	−23/64/−17	33% Rostral middle frontal 32% Superior frontal 19% Lateral orbitofrontal 12% Medial orbitofrontal	Left	2375	0.01^b^
Self-Concept	positive	−1/109/38	48% Superior frontal 23% Rostral middle frontal 15% Lateral orbitofrontal	Left	2582	0.004*^c^
DSM Hyp/Imp	positive	−29/83/61	34% Rostral middle frontal 31% Superior frontal 11% Medial orbitofrontal	Left	3194	0.01^d^
DSM Total Score	positive	−18/57/70	34% Superior frontal 20% Rostral middle frontal 14% Lateral orbitofrontal	Left	3410	0.018^e^

*MNI* Montreal Neurological Institute coordinate system; *DSM* diagnostic and statistical manual of mental disorders; *k* Cluster extent size.

* Significance determined at α = 0.006, corrected for 8 multiple comparisons based on the CAARS O:L subscales.

^a^TFCE value = 12486.34.

^b^TFCE value = 11557.97.

^c^TFCE value = 14231.74.

^d^TFCE value = 11418.91.

^e^ TFCE value = 10194.53.

### MPH group

**Fig. 6:**
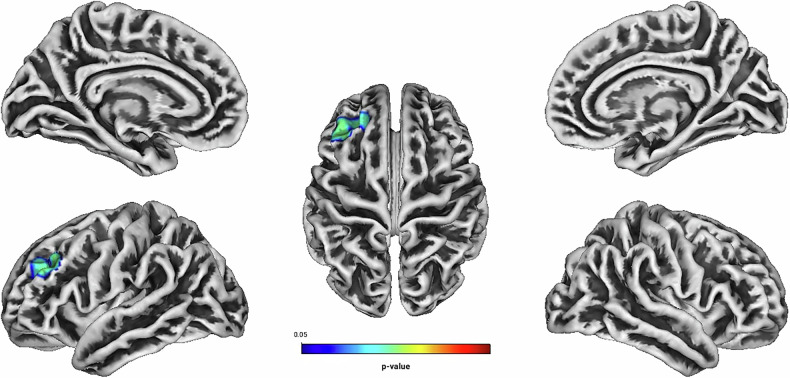
Negative association between cortical gyrification and CAARS O:L Hyperactivity Change (Weeks 0–13) for methylphenidate treatment. Cluster of significant positive correlation at p < 0.05 after TFCE, FWE-corrected combined peak-cluster level.

Among patients receiving MPH, Hyperactivity was negatively associated with GI in the left rostral middle frontal gyrus (see Table 3 and Fig. 6). Figure S1 illustrates the directionality of effects through trend lines, highlighting the relationship between GI and treatment outcomes across both treatment groups (see Supplemental Material). There was no significant group effect on ADHD-related symptom alleviation [F (8, 110) = 1.458, *p* = 0.181, partial η^2^ = 0.096, Wilk’s Λ = 0.904] nor were there any significant associations between Total Score, Inattention, Impulsivity, Self-Concept, and related DSM-Indexes and the GI in relation to treatment with MPH [all associated *TFCE* values < 4332.38, FWE-corrected peak-cluster level *p* > 0.184].

**Table 3:** Associations of cortical gyrification index and Hyperactivity Change (Weeks 0–13) in the methylphenidate subgroup (n = 121) measured by the CAARS O:L at p < 0.05, TFCE, FWE-corrected combined peak-cluster level.

Factor	Corre-lation	Coordinates of the maximum intensity voxel (x/y/z) MNI	Anatomical labelling	Hemisphere	*k*	*p*
Hyper activity	negative	−30/54/38	96% Rostral middle frontal	Left	375	0.036^a^

^a^TFCE value = 7978.3.

### GPT group

**Fig. 7:**
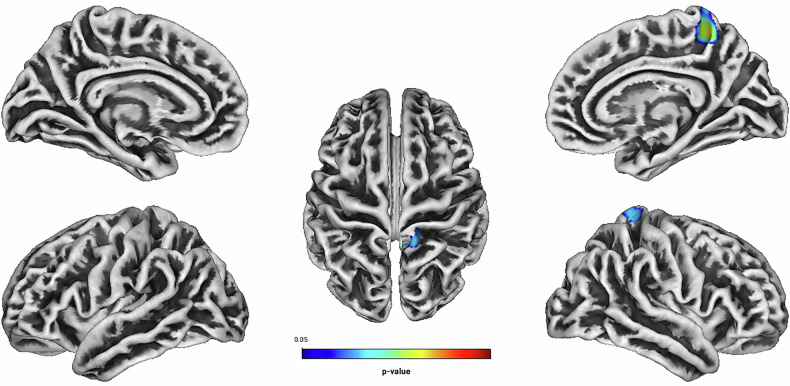
Negative association between cortical gyrification and CAARS O:L Inattention Change (Weeks 0–13) for the psychotherapy subgroup. Cluster of significant positive correlation at p < 0.05 after TFCE, FWE-corrected combined peak-cluster level.

In patients undergoing GPT, linear regression analyses showed a negative association between Inattention and GI located predominantly in the right precuneus and the paracentral gyrus (see Table 4 and Fig. 7). Figure S2 demonstrates the direction of effects using trend lines, highlighting the association between gyrification index and treatment outcomes across both treatment groups (see Supplemental Information). There was a significant group effect on overall symptom reduction [F (8, 110) = 2.476, *p* = 0.017, partial η^2^ = 0.153, Wilk’s Λ = 0.847]. CM was superior to GPT in reducing the total number of ADHD-related symptoms, symptoms of inattention and symptoms of impulsivity. There were no statistically significant relationships between Total Score, Hyperactivity, Impulsivity, Self-Concept and related DSM-Indexes and the GI [all associated *TFCE* values < 4077.74, FWE-corrected peak-cluster level *p* > 0.21].

**Table 4:** Associations of cortical gyrification index and Inattention Change (Weeks 0–13) in the psychotherapy subgroup (n = 121) measured by the CAARS O:L at p < 0.05, TFCE, FWE-corrected combined peak-cluster level.

Factor	Corre-lation	Coordinates of the maximum intensity voxel (x/y/z) MNI	Anatomical labelling	Hemisphere	*k*	*p*
Inattention	negative	14/-13/62	49% Precuneus 29% Paracentral 18% Superior parietal	Right	364	0.037^a^

^a^TFCE value = 7782.9.

## Discussion

While the importance of personalised treatment approaches is increasingly acknowledged, predicting treatment outcomes for adults with Attention-Deficit/Hyperactivity Disorder (ADHD) remains insufficiently explored. This study aimed to address this gap by investigating the prognostic value of cortical gyrification on initial treatment response following 12 weeks of treatment with GPT or CM combined with either MPH or PLA in adults with ADHD. Independent of treatment modality, we showed significant positive associations of cortical gyrification in frontal structures in the left hemisphere with overall ADHD symptom alleviation across three symptom dimensions. Treatment-specific effects showed superiority of CM in overall symptom reduction compared to GPT. Furthermore, distinct patterns of baseline gyrification predicted treatment response. Lower baseline gyrification in the (left) rostral middle frontal gyrus was associated with greater improvement in hyperactivity when treated with MPH, while reduced gyrification in the (right) precuneus, paracentral gyrus and superior parietal gyrus led to greater reduction of inattention when undergoing GPT.

In the primary analyses of the COMPAS study [52], we previously demonstrated that GPT and CM were similarly effective in reducing ADHD symptomatology over a 12-week period, with MPH showing superiority across almost all outcome domains. In our MRI subsample of the COMPAS cohort, these findings were not replicated. While a significant time effect indicated that all treatments contributed to a reduction in ADHD core symptomatology, group effects revealed a nuanced pattern. CM was more effective than GPT in reducing ADHD symptoms, while MPH did not outperform PLA. This discrepancy may be attributed to a strong placebo effect and the nature of CM that included face-to-face counselling activities. Potentially CM addressed individual patient needs better than group psychotherapy. Although MPH did not demonstrate statistical superiority over PLA, there was a notable trend indicating a greater effect of MPH compared to PLA.

To the best of our knowledge, no previous study has yet investigated the relationship between the GI of the cortex and subsequent treatment response independent of pharmacological or psychological intervention in ADHD. Hence, a direct comparison to existing literature is precluded. Moreover, there is only limited evidence for gyrification abnormalities in ADHD due to a scarcity in studies conducted. Pertaining to the evidence that is available and that was highlighted previously, Ambrosino et al. [39] reported decreased cortical folding in the left rostral middle frontal cortex and right pars opercularis, while Mous et al. [40] showed increased gyrification in the left medial temporal lobe in patients with ADHD. Conversely, Forde et al. [41], Shaw et al. [42], and Gharehgazlou et al. [43], did not report differences in either global or local gyrification between patients with ADHD and healthy controls. In addition, our research group could neither provide evidence for a difference in cortical gyrification between adult patients and controls [44]. Nevertheless, while there is only limited evidence highlighting gyrification abnormalities in ADHD, there may be evidence that left rostral middle frontal cortex gyrification may be shared by both ADHD and suboptimal treatment response [39].

While VBM and surface-based morphometric approaches are not directly comparable, our findings may be discussed in the context of previous studies suggesting that diminished grey matter tissue or cortical thickness in frontal structures is associated with poor treatment response [66, 67]. In a naturalistic study with 107 children diagnosed with ADHD, Shaw et al. [67] reported that a thinner left medial prefrontal cortex at baseline was associated with less favourable clinical outcomes, while greater cortical thickness in this region was linked to greater symptom reduction. Parlatini et al. [66] showed that adult non-responders to MPH treatment for 2 months had lower cortical volume and surface area in the right lateral orbital frontal cortex, the right rostral middle frontal gyrus and right caudal middle frontal gyrus in comparison to responders. Interestingly, Yang et al. [68] highlighted that the left dorsolateral prefrontal cortex and the medial prefrontal cortex showed distributed patterns of functional connectivity across the entire brain that were altered by 3-week psychostimulants and were associated with symptom improvement. Nevertheless, given the scarcity of literature on cortical gyrification in ADHD, particularly predicting treatment success, further research is imperative to expand our understanding of frontal cortical gyrification in ADHD and its role in predicting treatment success. This need for further investigation gains additional relevance in the light that we show conflicting treatment-specific results in response to MPH and GPT treatment.

Lower baseline cortical gyrification in the rostral middle frontal gyrus was negatively associated with reduction in symptoms of hyperactivity when treated with MPH. Though initially counterintuitive, this observation aligns with concept of an inverted-U-shaped dose-response curve for MPH [69, 70], implicating that the same quantity of MPH may lead to domain specific symptom alleviation but may increase symptom burden in another. Thus, increased cortical gyrification in frontal structures on the left hemisphere may predispose patients to experience overall symptom alleviation in response to MPH, but increased cortical gyrification in the left rostral middle frontal gyrus may instead exacerbate hyperactivity symptoms.

Baseline cortical gyrification in the (right) precuneus, paracentral gyrus und superiorparietal cortex was also negatively associated with symptoms of inattention in patients undergoing GPT. We previously showed a negative association between cortical gyrification in a cluster combining the (right) pre-, para-, and postcentral gyri with extension to the precuneus and symptoms of inattention [50]. Here we extend on this finding, showing that cortical gyrification of the (right) precuneus and paracentral gyrus is not only negatively correlated with symptoms of inattention, but that it may also lead to patients receiving less symptom alleviation from psychotherapy. Given that psychological interventions can influence both brain function [31] and -structure [33–35], patients with a less structurally complex (right) precuneus and paracentral gyri prior to therapy may exhibit greater plasticity. Consequently, patients may achieve more substantial improvements in attention compared to those with higher cortical complexity and lower plasticity. Nevertheless, given the limited body of evidence surrounding cortical gyrification in adults with ADHD and its predictive value of treatment success, all hypotheses about the interaction of MPH and GPT in combination with cortical gyrification to predict treatment response warrant further investigation and validation.

Still, our findings fit the hypothesis that abnormal brain gyrification, likely as a consequence of a more severe disruption of neurodevelopmental processes, may predispose patients to derive less from treatment, dependent and independent of treatment that is being received. The most prominent cortical folding changes occur during the third trimester of pregnancy, but gyrification continues to change in different ways throughout the first decades of life [71]. Disruption of neurodevelopmental processes caused by genetic or environmental influences may lead to abnormal cortical gyrification predisposing individuals to psychiatric conditions [72]. Hence, cortical gyrification may not only serve as a marker of integrity of normal cortical development [36, 38, 73, 74], but may also hold promise in characterising subgroups of patients with low response to treatments [36].

Cortical gyrification, especially in frontal regions, may offer valuable insights, as greater gyrification in adults’ bilateral medial and superior frontal cortex has been linked to better working memory and mental flexibility task performance [75, 76]. Gregory et al. [77] showed that in healthy adults, higher general cognitive ability was positively associated with local gyrification index in several brain regions, including the lateral prefrontal cortex, cingulate, insular cortices, inferior parietal lobule, temporoparietal junction regions, and fusiform gyrus. Specifically, the lateral prefrontal cortex appears as a pivotal region, as its functional connectivity and structure have been implicated in general cognitive control [78], overall intelligence [79], and various executive functions [80]. Pertaining to our results of cortical gyrification in frontal regions of the left hemisphere, one potential interpretation is that individuals with higher cognitive ability might derive greater benefit from therapy. Pharmaco- and/or psychotherapy may empower patients to leverage cognitive ability to address life circumstances that were previously challenging due to lack of guidance or ability. While personal agency significantly influence psychological well-being throughout life [81], neurocognition has also been associated with treatment response in ADHD [82, 83]. Neurocognition and frontal cortical gyrification as treatment predictors would be of great importance for the identification of patients with suboptimal response to standard treatments.

### Limitations

Given the scarcity of studies exploring treatment response in adults with ADHD, it is currently premature to draw definitive conclusions. A cohesive understanding of abnormalities in cortical gyrification in ADHD patients has yet to emerge. Anatomical studies on treatment response have produced heterogeneous results, reporting instances of hyper-gyrification, hypo-gyrification or a complex combination affecting both the cortex and subcortical structures [25, 84]. Consequently, validating our findings with previous reports is challenging. Moreover, translating these findings clinical practice remains inherently challenging, as the utility of cortical gyrification as a predictive biomarker is constrained by considerable interindividual variability in symptom manifestations, developmental neuroplasticity, and the profound heterogeneity of ADHD’s neurobiological architecture, highlighting the need for replication in larger and more diverse cohorts before meaningful clinical implementation can be achieved. Additionally, although all results have been corrected for multiple comparison within the analysis, no correction for multiple testing across all analyses was applied. After implementing correction for multiple testing across all analyses, only the cluster identified for self-concept retains statistical significance. Finally, while TFCE statistical approaches have demonstrated increased sensitivity in detecting significant results, the method has also been shown to induce more spatial bias [85].

### Future directions

Further investigation into treatment response in adults with ADHD is essential and warranted to validate our findings. Multimodal studies integrating functional data with structural abnormalities hold promise for gaining a more comprehensive understanding of the intricate relationship between cortical and functional irregularities and symptom manifestation and persistence. In this context, machine learning approaches may prove valuable in identifying predictive cortical markers prior to treatment initiation, thereby improving treatment stratification, reducing unnecessary delays in administering individualised treatment, and mitigate patient discouragement. Prolonged treatment durations coupled with multiple longitudinal anatomical scans may further aid in revealing additional biomarkers associated with treatment response. Additionally, exploring the interplay of neurocognition with (in)dependent treatment response could inform more personalised treatment strategies. Investigating the potential role of genetic markers in moderating treatment outcomes may also further enhance our understanding of treatment response heterogeneity in ADHD. Lastly, longitudinal studies tracking treatment response over extended periods and across different stages of adulthood could elucidate the long-term effectiveness and durability of interventions.

## Supplementary information

Supplemental Material

## Data Availability

The data will be made available from the corresponding author [JL] upon reasonable request. The data is not publicly available due to privacy and ethical restrictions.
