# Supplementary material for: Cortical gyrification predicts initial treatment response in adults with ADHD
Source: Transl Psychiatry. 2025 Oct 17;15:406. doi: 10.1038/s41398-025-03681-0 (PMC12534422; doi:10.1038/s41398-025-03681-0)
Supplement: Supplementary file 1 — Supplemental Material [file 41398_2025_3681_MOESM1_ESM.docx]

**SUPPLEMENTAL MATERIAL**

**Table S1.** Summary of inclusion and exclusion criteria

| Inclusion criteria | Written informed consent  Sufﬁcient German language skills  Age 18–60 years, inclusive  Diagnosis of ADHD according to DSM-IV criteria  Chronic course of ADHD symptoms from childhood  to adulthood, and Wender-Utah Rating Scale short  version ≥ 30  No pathological abnormality detected on physical examination,   routine blood testing (blood count, renal, hepatic, and thyroid function), ECG, and EEG  Baseline assessment within 7 days, able to begin  treatment within 14 days |
| --- | --- |
| Exclusion criteria | IQ < 85 (Multiple-Choice Vocabulary Test < 17)  Schizophrenia, bipolar disorder, borderline  personality disorder, antisocial personality disorder,  suicidal or self-injurious behavior, autism, motor  tics, Tourette’s syndrome  Substance abuse/dependence within 6 months prior  to screening (episodic abuse is not an exclusion  criterion); positive drug screening  Neurological diseases, seizures, glaucoma, diabetes  mellitus, hyperlipidemia, uncontrolled arterial  hypertension, angina pectoris, tachycardia  arrhythmia, arterial occlusive disease  Previous stroke  Current eating disorder (bulimia and anorexia), low  weight (BMI < 20, later protocol amendment  BMI < 19)  Pregnancy (current or planned) or breast-feeding; no  reliable contraception (Pearl Index < 1%)  Participation in another clinical trial (up to 3 months  prior)  Treatment with stimulants or ADHD-speciﬁc  psychotherapy in the 6 months prior to study  inclusion  Known MPH intolerance  Treatment with antidepressants (e.g., SSRI, TCA),  NRI (e.g., atomoxetine), bupropion, neuroleptic  medication, theophylline, amantadine,  anticoagulants derived from coumarin,  phenylbutazone, antacids, and alpha-adrenergic  agonists (e.g., clonidine) in the 2 weeks prior to  baseline assessment (T1)  Treatment with ﬂuoxetine, monoamine oxidase  inhibitors in the 4 weeks prior to baseline  assessment (T1)  Refusal to comply with study requirements  Refusal to comply with study requirements |

*BMI,* body mass index; *MPH,* methylphenidate; *SSRI,* selective serotonin reuptake inhibitor; *TCA,* tricyclic antidepressant; *NRI,* norepinephrine reuptake inhibitor

**Table S2**

*Baseline symptomatology across the MPH and PLA treatment groups*

| Measure | MPH (*n*=58) | PLA (*n*=63) | F | *p* |
| --- | --- | --- | --- | --- |
| Total Score | 98.57 ± 29.2 | 95.87 ± 30.2 | .256 | .614 |
| Inattention / Memory Problems | 20.19 ± 6.9 | 18.48 ± 7.75 | 1.81 | .181 |
| Hyperactivity/ Restlessness | 16.36 ± 7.94 | 16.06 ± 7.8 | .045 | .832 |
| Impulsivity | 15.84 ± 6.9 | 17.19 ± 6.83 | 1.19 | .278 |
| Problems with Self-Concept | 10.88 ± 3.91 | 9.76 ± 4.5 | 2.02 | .158 |
| DSM Inattention / Memory Problems | 15.57 ± 5.25 | 14.54 ± 5.78 | 1.05 | .307 |
| DSM Hyperactivity/Impulsivity | 9.36 ± 5.5 | 9.94 ± 5.6 | .293 | .589 |
| DSM Total Score | 24.91 ± 8.76 | 24.48 ± 9.09 | .083 | .773 |

**Table S3**

*Baseline symptomatology across the GPT and CM treatment groups*

| Measure | GPT (*n*=59) | CM (*n*=62) | F | *p* |
| --- | --- | --- | --- | --- |
| Total Score | 92.1 ± 31.25 | 101.98 ± 27.4 | 3.41 | .067 |
| Inattention / Memory Problems | 18.34 ± 7.94 | 20.21 ± 6.74 | 2.16 | .145 |
| Hyperactivity/ Restlessness | 15.73 ± 7.43 | 16.66 ± 8.24 | .442 | .507 |
| Impulsivity | 15.86 ± 7.23 | 17.19 ± 6.51 | 1.06 | .305 |
| Problems with Self-Concept | 9.54 ± 4.41 | 11.02 ± 3.99 | 3.39 | .068 |
| DSM Inattention / Memory Problems | 14.2 ± 6.3 | 15.82 ± 4.59 | 2.6 | .110 |
| DSM Hyperactivity/Impulsivity | 9.2 ± 5.26 | 10.1 ± 5.76 | .842 | .361 |
| DSM Total Score | 23.41 ± 9.42 | 25.9 ± 8.27 | 2.445 | .121 |

**Table S4**

*Psychometric data across the full patient sample and subsamples with analysis of group differences over time*

| CAARS O:L | Baseline  Mean ± SD | Week 13  Mean ± SD | Change  Mean ± SD | F | *p* |
| --- | --- | --- | --- | --- | --- |
| Total Score |  |  |  |  |  |
| Total Sample *(N=121)* | 97.17 ± 29.63 | 79.18 ± 30.24 | 17.98 ± 27.38 |  |  |
| MPH (*n*=58) | 98.57 ± 29.2 | 77.98 ± 27.57 | 20.59 ± 26.01*** | 1.13 | .291 |
| PLA (*n*=63) | 95.87 ± 30.2 | 80.29 ± 32.7 | 15.59 ± 28.59*** |  |  |
| GPT (*n*=59) | 92.1 ± 31.25 | 79.98 ± 30.88 | 12.12 ± 28.84*** | 5.86 | .017* |
| CM (*n*=62) | 101.98 ± 27.4 | 78.42 ± 29.86 | 23.56 ± 24.89*** |  |  |
| Inattention  /Memory Problems |  |  |  |  |  |
| Total Sample *(N=121)* | 19.3 ± 7.38 | 15.63 ± 7.46 | 3.67 ± 6.25 |  |  |
| MPH (*n*=58) | 20.19 ± 6.9 | 15.91 ± 6.76 | 4.28 ± 5.99*** | 1.33 | .252 |
| PLA (*n*=63) | 18.48 ± 7.75 | 15.37 ± 8.09 | 3.11 ± 6.48*** |  |  |
| GPT (*n*=59) | 18.34 ± 7.94 | 16.05 ± 8.15 | 2.29 ± 6.11** | 6.83 | .01* |
| CM (*n*=62) | 20.21 ± 6.74 | 15.23 ± 6.78 | 4.98 ± 6.14*** |  |  |
| Hyperactivity  /Restlessness |  |  |  |  |  |
| Total Sample *(N=121)* | 16.21 ± 7.84 | 13.22 ± 7.17 | 2.98 ± 6.09 |  |  |
| MPH (*n*=58) | 16.36 ± 7.94 | 12.95 ± 7.05 | 3.41 ± 5.67*** | .57 | .454 |
| PLA (*n*=63) | 16.06 ± 7.8 | 13.48 ± 7.31 | 2.59 ± 6.46** |  |  |
| GPT (*n*=59) | 15.73 ± 7.43 | 13.1 ± 14.03 | 2.63 ± 6.5** | .39 | .532 |
| CM (*n*=62) | 16.66 ± 8.24 | 13.34 ± 7.35 | 3.32 ± 5.7*** |  |  |
| Impulsivity |  |  |  |  |  |
| Total Sample *(N=121)* | 16.55 ± 6.87 | 13.54 ± 6.3 | 3.01 ± 6.22 |  |  |
| MPH (*n*=58) | 15.84 ± 6.9 | 12.81 ± 6.62 | 3.03 ± 6.2*** | <.01 | .965 |
| PLA (*n*=63) | 17.19 ± 6.83 | 14.21 ± 5.97 | 2.98 ± 6.28*** |  |  |
| GPT (*n*=59) | 15.86 ± 7.23 | 14.03 ± 6.24 | 1.83 ± 7.04* | 4.17 | .043* |
| CM (*n*=62) | 17.19 ± 6.51 | 13.06 ± 6.38 | 4.13 ± 5.14*** |  |  |
| Problems with   Self-Concept |  |  |  |  |  |
| Total Sample *(N=121)* | 10.29 ± 4.24 | 8.96 ± 4.87 | 1.34 ± 3.67 |  |  |
| MPH (*n*=58) | 10.88 ± 3.91 | 8.93 ± 4.65 | 1.95 ± 4.05*** | 3.14 | .079 |
| PLA (*n*=63) | 9.76 ± 4.5 | 8.98 ± 5.1 | .78 ± 3.22 |  |  |
| GPT (*n*=59) | 9.54 ± 4.41 | 8.61 ± 5.3 | .93 ± 3.68 | 1.43 | .234 |
| CM (*n*=62) | 11.02 ± 3.99 | 9.29 ± 4.44 | 1.73 ± 3.67*** |  |  |

| DSM Inattention   /Memory Problems |  |  |  |  |  |
| --- | --- | --- | --- | --- | --- |
| Total Sample *(N=121)* | 15.03 ± 5.53 | 11.74 ± 5.2 | 3.29 ± 5.25 |  |  |
| MPH (*n*=58) | 15.57 ± 5.25 | 11.72 ± 4.82 | 3.84 ± 5.19*** | 1.39 | .241 |
| PLA (*n*=63) | 14.54 ± 5.78 | 11.76 ± 5.57 | 2.78 ± 5.3*** |  |  |
| GPT (*n*=59) | 14.2 ± 6.3 | 12.42 ± 5.79 | 1.78 ± 5.1* | 10.97 | .001** |
| CM (*n*=62) | 15.82 ± 4.59 | 11.1 ± 4.53 | 4.73 ± 5.02*** |  |  |
| DSM Hyperactivity  /Impulsivity |  |  |  |  |  |
| Total Sample *(N=121)* | 9.66 ± 5.52 | 7.88 ± 5.01 | 1.78 ± 4.1 |  |  |
| MPH (*n*=58) | 9.36 ± 5.5 | 7.81 ± 5.15 | 1.55 ± 3.96** | .23 | .631 |
| PLA (*n*=63) | 9.94 ± 5.6 | 7.95 ± 4.91 | 1.98 ± 4.25*** |  |  |
| GPT (*n*=59) | 9.2 ± 5.26 | 7.56 ± 4.09 | 1.64 ± 4.38** | .23 | .634 |
| CM (*n*=62) | 10.1 ± 5.76 | 8.19 ± 5.77 | 1.9 ± 3.84*** |  |  |
| DSM Total Score |  |  |  |  |  |
| Total Sample *(N=121)* | 24.69 ± 8.9 | 19.63 ± 8.45 | 5.06 ± 8.06 |  |  |
| MPH (*n*=58) | 24.91 ± 8.76 | 19.53 ± 8.07 | 5.38 ± 7.87*** | .27 | .607 |
| PLA (*n*=63) | 24.48 ± 9.09 | 19.71 ± 8.84 | 4.76 ± 8.28*** |  |  |
| GPT (*n*=59) | 23.41 ± 9.42 | 19.98 ± 7.81 | 3.42 ± 8.46** | 5.59 | .02* |
| CM (*n*=62) | 25.9 ± 8.27 | 19.29 ± 9.06 | 6.61 ± 7.38*** |  |  |

** p < .05 ** p < .01 *** p < .001
Note. F* statistic for group differences over time: MPH vs. PLA; GPT vs. CM

**Figure S1**

*Flowchart of participant selection and retention throughout the imaging study.*


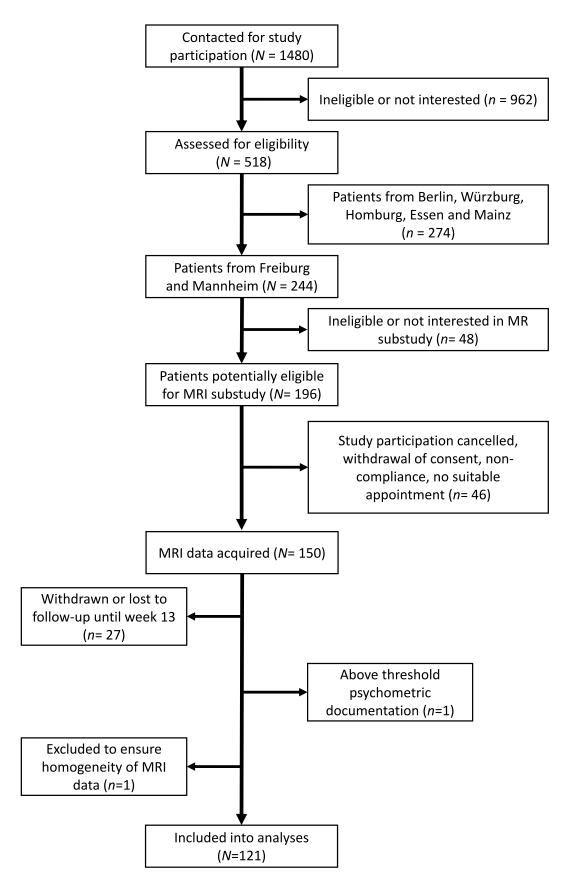


**Figure S2**

*Directionality of the effects between cortical gyrification and hyperactivity treatment outcomes following 12 weeks of methylphenidate treatment compared to placebo*

**Figure S3**

*Directionality of the effects between cortical gyrification and inattention treatment outcome following 12-weeks of group psychotherapy compared to clinical management*
